# Linking NCBI to Wikipedia: a wiki-based approach

**DOI:** 10.1371/currents.RRN1228

**Published:** 2011-03-31

**Authors:** Roderic D. M. Page

**Affiliations:** Institute of Biodiversity, Animal Health and Comparative Medicine College of Medical, Vetinary and Life Sciences University of Glasgow Glasgow G12 8QQ, UK

## Abstract

The NCBI Taxonomy underpins many bioinformatics and phyloinformatics databases, but by itself provides limited information on the taxa it contains. One readily available source of information on many taxa is Wikipedia. This paper describes iPhylo Linkout, a Semantic wiki that maps taxa in NCBI's taxonomy database onto corresponding pages in Wikipedia. Storing the mapping in a wiki makes it easy to edit, correct, or otherwise annotate the links between NCBI and Wikipedia. The mapping currently comprises some 53,000 taxa, and is available at http://iphylo.org/linkout. The links between NCBI and Wikipedia are also made available to NCBI users through the NCBI LinkOut service.

## Introduction 

One of the great challenges of phyloinformatics is linking together information on phylogenies, taxa, genomes, specimens, and publications [1]. There are a plethora of biodiversity databases [2], but a dearth of services that map identifiers across these databases. Identifiers from one database are rarely reused by other databases [3], and even databases hosted by the same organisation may be disconnected [4]. As a consequence, much of the fundamental data of biodiversity is trapped in separate silos. One way to address the lack of integration between biodiversity databases is to develop tools and services that map identifiers between these databases. 

Stein [5] recognised two classes of bioinformatics resources: "knuckles" and "nodes". Nodes represent databases containing information on a specific topic (for example, genes), whereas knuckles link records in different databases (i.e., this gene in database A corresponds to that gene in database B). This paper describes the construction one such tool, a biodiversity knuckle that maps identifiers between two major resources, the NCBI taxonomy database [6] and Wikipedia [7]. The goal is to provide a simple way for users of the NCBI taxonomy database to quickly discover basic information about a taxon, such as what it looks like, where it is found, and some basic biology. In addition, the mapping makes it easy to add annotations to databases that make use of NCBI identifiers, including phylogeny databases such as TreeBASE [8] [9]. 

### NCBI and Wikipedia 

The NCBI taxonomy database provides the taxonomy underlying GenBank [10]. Any sequence submitted to GenBank gets a NCBI taxonomy identifier (hereafter referred to as the "tax_id") and this tax_id is reused across multiple databases derived from GenBank, making it the default taxonomic identifier for most bioinformatics projects. Within the phylogenetics community both TreeBASE and PhyLoTa [11] [12] make use of use NCBI tax_ids. 

In the ten years since its launch [13] Wikipedia has become the Internet's default resource for basic information about many topics. For taxonomic names Wikipedia is an order of magnitude more likely to be the top result in a Google search than any other site on the Internet [14]. It is also central to many efforts to create a web of data, such as DBpedia [15] [16] and Freebase [17]. Wikipedia is also being used by organisations that want to display their own content in a broader context, such as the BBC's reuse of Wikipedia identifiers and content in the BBC Wildlife Finder [18] [19]. In addition to being a source of basic information, Wikipedia is increasingly playing a role in capturing annotations of biological data. The GeneWiki project [20] has created Wikipedia pages for each human gene family, with the goal of engaging at least some Wikipedia users in the task of enriching these pages. Reflecting the increasing engagement of the scientific community with Wikipedia, the journal *RNA Biology* now requires authors of articles on RNA families to create a corresponding article in Wikipedia [21]. 

Given the centrality of the NCBI taxonomy database within bioinformatics [22], and the growth  of information about taxa in Wikipedia [14], it seems natural to link these two databases together. This would enable users of the NCBI database, for example, to find out more about a taxon by clicking on the link to Wikipedia, and Wikipedia users could click on the link to the corresponding NCBI taxon to discover information about that taxon's genomics. Other databases that use identifiers from either NCBI or Wikipedia could also make use of the mapping. For example, wherever possible TreeBASE includes NCBI tax_ids for taxa it contains. Given a mapping from NCBI tax_ids to Wikipedia pages, it would be straightforward to extract taxon images from Wikipedia and create illustrations of phylogenies from, for example, TreeBASE, annotated with images of the taxa contained in those phylogenies.

## Linking databases 

There are several ways we could link two databases together. One is to create reciprocal links in both databases, that is, the NCBI page for a taxon would contain a link to the Wikipedia page, and visa versa. Although having the original databases manage the links has obvious advantages, there may be reasons for devolving this task to a third party. Maintaining and updating links requires work, and those managing the database may not have sufficient resources to provide this service. They may also feel that the links are beyond the intended scope of the database.

Having the links stored in a third party database (the "knuckle") enables the links to be edited and annotated. If the rationale for a particular link requires explanation (for example, a link based on taxonomic synonymy), or if either of the source databases contain errors (or controversial records), these can be flagged in the knuckle database. This enables annotations on databases in cases where those managing the data may be reluctant to let users modify the primary data [23]. Additional information, such as links to original taxon descriptions or nomenclatural decisions could also be added, enriching the mapping between the two databases. 

A further reason for an intermediate service is to provide programmatic access to the mapping. NCBI provides utilities to retrieve LinkOut resources [24], but retrieving the reverse links from a Wikipedia page requires text mining to extract URLs. A third party service could circumvent this difficulty by providing an API to access the mapping directly. 

## Constructing the mapping 

The mapping between NCBI was initially based upon the 18 June 2009 SQL dump of Wikipedia and a dump of the NCBI taxonomy database obtained on 29 September 2009. The NCBI taxonomy database is provided as a series of delimited text files, which can be readily loaded into a MySQL database. Taxon pages were extracted from Wikipedia by loading the Wikipedia SQL dump into a MySQL database and searching for pages that included the Taxobox template (http://en.wikipedia.org/wiki/Template:Taxobox). The Taxobox template records basic facts about the taxon, such as its name, taxonomic classification, and optional details of synonymy, authorship, geographic range, and an image of one or more members of the taxon. Below is an example from the page for the Giant freshwater stingray http://en.wikipedia.org/wiki/Giant_freshwater_stingray:

{{Taxobox 

| name = Giant freshwater stingray

| image = Himantura chaophraya.jpg

| image_width = 240 px

| status = VU | status_system = IUCN3.1

| status_ref = <ref name="iucn">{{IUCN2008 |assessors=Shark Specialist Group |year=2000 |id=10048 |title=Himantura chaophraya |downloaded=March 24, 2009}}</ref>

| regnum = [[Animalia]]

| phylum = [[Chordata]]

| classis = [[Chondrichthyes]]

| subclassis = [[Elasmobranchii]]

| ordo = [[Myliobatiformes]]

| familia = [[Dasyatidae]]

| genus = ''[[Himantura]]''

| species = '''''H. chaophraya'''''

| binomial = ''Himantura chaophraya''

| binomial_authority = Monkolprasit & Roberts, 1990

}} 

Because this template is standardised it is relatively straightforward to parse it and extract details about the taxon that is the topic of the page.

### Mapping NCBI taxa to Wikipedia pages

The initial mapping was constructed by extracting the scientific name of the taxon that was the topic of each Wikipedia page, then finding a match for this in the NCBI taxonomy database. If NCBI and Wikipedia use the same name for the same taxon then in most cases the mapping is straightforward, we simply link the two records. However, because plant and animal names are governed by separate codes of nomenclature the same name may be used for a plant and an animal, such as *Abronia* (a genus of lizard and plant) and *Rondeletia* (a genus of fish and a genus of plant). To minimise these errors the GenBank "division_id" in NCBI and the "regnum" value in the Wikipedia Taxobox was used to distinguish between plants and animal names.

Duplication of names can also result from the way NCBI assigns names to nodes in its taxonomical hierarchy. For example, the genus *Rana* includes several subgenera, including *Rana (Rana)*. NCBI uses just the subgenus name for the subgeneric nodes, hence tax_ids 8399 and 121175 both bear the name "Rana".

Both the NCBI taxonomy and Wikipedia have support for synonyms, that is, different names for the same taxon. In the NCBI database there may be multiple names that have the same tax_id, but only one will be regarded as the accepted scientific name. In Wikipedia a synonym may have its own page in the form of a "redirect" page which automatically sends the user to the page with the name that Wikipedia regards as the accepted name.

**Figure:**
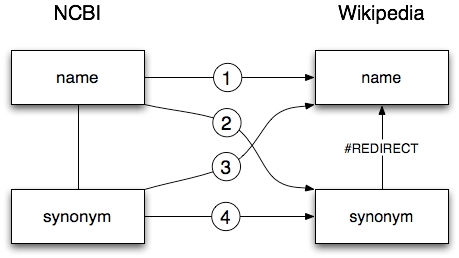
**Fig. 1.** Different possible mappings between taxon records in NCBI and Wikipedia pages. Both databases may include synonyms. In the case of Wikipedia a synonym may be a separate page that automatically redirects the user to the actual page for a taxon. 

If both NCBI and Wikipedia agree on the name of a taxon then the mapping is straightforward and is indicated by ➀ in Fig. 1. In other cases we may have to use synonyms to link the two databases. For example, at the time of writing the Abo bat has the accepted name *Chalinolobus poensis *in the NCBI taxonomy, but there is no corresponding page in Wikipedia that has *Chalinolobus poensis* as the accepted name. However, there is a Wikipedia page for *Chalinolobus poensis, *which is a redirect to the page for the Abo bat (for which Wikipedia gives the accepted name as *Glauconycteris poensis*). This is an example of a type ➁ mapping in Fig. 1, where the link from NCBI to Wikipedia is made via a Wikipedia redirect page. In the case of the Abo bat, NCBI lists *Glauconycteris poensis* as a synonym, so we could also discover this link via a type ➂ mapping (NCBI synonym to Wikipedia page). The last type of mapping, ➃, is where NCBI and Wikipedia disagree on the accepted name for a taxon, but a synonym in NCBI corresponds to a redirect page in Wikipedia.

### Gregg's paradox

Gregg [25] argued that many traditional biological classifications contain redundancy, resulting in taxa that have identical content occurring more than once in the classification (this has become known as "Gregg's paradox" [26]). Previously I [14] used the example of the aardvark (*Orycteropus afer*) to illustrate this, where the Wikipedia pages for Tubulidentata, Orycteropodidae, *Orycteropus*, and *Orycteropus afer* were one and the same page, because there is only a single extant species of *Orycteropus* *afer*, and it is the sole extant member of the family Orycteropodidae, and the order Tubulidentata. It is only when we add fossil aardvarks that we can create unique content for these other taxonomic ranks (and such pages have since been added to Wikipedia). However, there are still cases where more than one NCBI taxon will map to the same Wikipedia page (e.g., a monotypic genus and its sole species). In iPhylo Linkout only the lowest taxonomic rank is mapped (e.g., for a monotypic genus the species would be mapped, but not the genus).

## Implementation 

The mapping between NCBI and Wikipedia is stored in a wiki at http://iphylo.org/linkout. This makes it easy to edit, add to, or annotate the mapping. The wiki software used is the Semantic Mediawiki extension [27] to Mediawiki [28] (the later is the software that runs Wikipedia). Semantic Mediawiki adds several key features to Mediawiki, including support for semi-structured data, web forms, a simple query language, and exporting individual pages in RDF [29]. 

### Storing the mapping

The iPhylo Linkout wiki was populated using the Mediawiki API. Batch scripts read the NCBI taxonomy data dump, and called the Mediawiki API to create a wiki page for each NCBI tax_id, the page name being the prefix "Ncbi:" followed by the tax_id. This naming convention makes it easy to refer to any NCBI taxon by simply appending the tax_id to http://iphylo.org/link out/Ncbi:. To enable easy retrieval of pages by taxon name, pages were also created for each taxon name in the NCBI taxonomy. These pages automatically redirect the user to the corresponding "Ncbi:tax_id" page. In cases where the same name may refer to more than one taxon (e.g., *Morus* is a genus of plant and a genus of bird) the wiki page is a disambiguation page which lists the different "Ncbi:tax_id" pages with the same name.

Each taxon page in iPhylo Linkout uses Mediawiki's template language to describe the taxon and the mapping to Wikipedia. A template in Mediawiki is essentially a set of key-value pairs, where the "key" is the name of a data field and the "value" is the content of that field. The key-value pairs used in iPhylo Linkout are shown in Table 1.

**Table 1.** Key-value pairs used to describe the mapping between NCBI and Wikipedia.

**Table:** 

Key	Value
Key	Value
ncbi	NCBI tax_id for taxon
name	taxon name in the NCBI database
homonym	yes/no flag if name is a homonym
wikipedia_en	title of corresponding page in English language Wikipedia
wikipedia_en_id	page id of corresponding page in English language Wikipedia
wikipedia_en_snippet	first few lines of Wikipedia page
wikipedia_image	name of image of taxon that appears in Taxobox in Wikipedia page
rank	rank of taxon (e. g., species, genus, etc.)
division_id	GenBank division
parent	tax_id (with prefix “ncbi:”) of parent taxon in NCBI classification
synonyms	semicolon delimited list of names GenBank regards as synonyms of this taxon

The code for a wiki page from the mapping (http://iphylo.org/linkout/Ncbi:87131) is shown below (with some values truncated for clarity):

{TaxonConcept

|ncbi=87131

|name=Himantura chaophraya

|homonym=No

|wikipedia_en=Giant_freshwater_stingray

|wikipedia_en_id=8224153

|wikipedia_en_snippet=The giant freshwater stingray, Himantura chaophraya...

|wikipedia_image=Himantura_chaophraya.jpg

|rank=species

|division_id=10

|parent=ncbi:86362

|synonyms=Himantura chaophraya Monkolprasit and Roberts, 1990;Himantura chaophyraya;freshwater whipray

}}

If a NCBI taxon has a matching page in Wikipedia, the corresponding page in iPhylo Linkout includes the title of the Wikipedia page, the corresponding numeric page id, a snippet of text from Wikipedia (typically the first few lines of text on the page), and the name of the image file (if any). The Wikipedia page id is stored as the name of a Wikipedia page may change over time, whereas the page id is stable (Freebase [30] takes the same approach to storing links to Wikipedia). The id for page in Wikipedia can be obtained by viewing the XML for a Wikipedia page using the "Special:Export" feature.

Although primarily intended to provide a simple mapping between NCBI and Wikipedia, iPhylo Linkout uses the query language provided by Semantic Mediawiki to provide a way to navigate around the NCBI taxonomic hierarchy. For each taxon, links to the parent and all the child taxa are shown, together with thumbnails of images of those taxa (if available) (Fig. 2).

**Figure:**
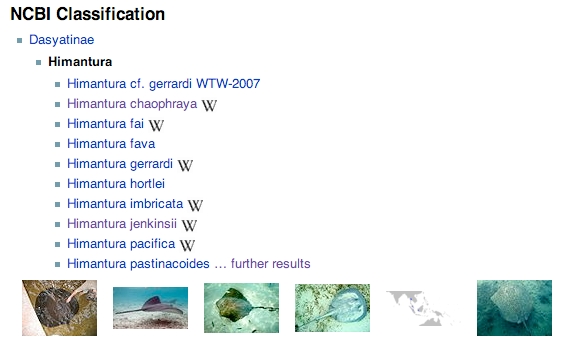
**Fig. 2.** Simple display of the parent and child taxa for the genus *Himantura* in iPhylo Linkout (see http://iphylo.org/linkout/Ncbi:86362).

### Citations

In addition to information about taxa, the NCBI taxonomy database dump contains bibliographic information in a file called citations.dmp. Each record in this file has a unique identifier (the "cit_id") and is linked to one or more tax_ids. Many, but not all, of these bibliographic records include PubMed identifiers (PMID). Records that lacked PMIDs were parsed and the bioGUID OpenURL service [31] [32] used to find alternative identifiers such as DOIs. Because a single citation record may be linked linked to multiple taxa, a page in the iPhylo Linkout wiki was created for each citation record, the page name being the prefix "Citation:" followed by the cit_id, e.g. http://iphylo.org/linkout/Citation:4306. These citation pages were then included in the wiki page for the corresponding tax_id(s) using Mediawiki's transclusion mechanism (see http://iphylo.org/linkout/Ncbi:77658 for an example). This has the advantage that any edits made to the citation page (for example, adding a DOI) are automatically reflected in all the taxon pages that include that citation.

### Data export

Semantic Mediawiki supports exporting individual pages in RDF (see Fig. 3). There are numerous vocabularies that could be used to express information in RDF, iPhylo Linkout uses the most general vocabulary wherever possible. Following Uniprot [33] [34] the relationship between child and parent taxa is described using the rdfs:subClassOf relationship (where "rdfs" refers to the RDF Schema namespace [35]). NCBI tax_ids already exist in Linked Data space in both Uniprot and the Bio2RDF project [36] [37], so the RDF export asserts that the entries in iPhylo Linkout out refer to the same entities as the Uniprot and bio2rdf URIs using the owl:sameAs predicate (where "owl" is the Web Ontology Language namespace [38]). The link to Wikipedia, if it exists, is asserted using the equivalent DBPedia URI (the Wikipedia page name prefixed by "http://dbpedia.org/resource/"), together with URLs based on both the Wikipedia page name and the page id. Page names are more widely used, but tend to be less stable than the page ids. If the Wikipedia page includes an image of the taxon, this information included in the RDF using the FOAF vocabulary [39]. The name used by NCBI for the taxon, and its rank are specified using the TDWG LSID vocabularies [40]. 

**Figure:**
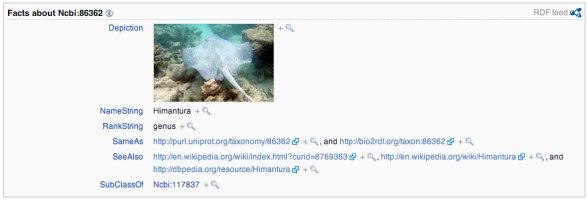
**Fig. 3.** RDF for the iPhylo Linkout page for NCBI taxon tax_id 87131 (*Himantura chaophraya*).

An advantage of providing RDF is that users wanting to retrieve details about the mapping between a NCBI taxon and Wikipedia can do so using standard tools for XML parsing, rather than attempt to parse the Mediawiki template.

## Integrating the mapping into NCBI and Wikipedia 

An limitation of having the mapping stored in a third party database is that users of NCBI and Wikipedia may be unaware of the existence of that third database. Ideally the mapping could be included in both "node" databases so that users can easily go from one database to another. 

NCBI LinkOut [24] provides a mechanism to link NCBI database records to those in other databases. These links are provided by third parties, and are shown, for example, on the page for each NCBI taxon. The NCBI-Wikipedia mapping described here is made available through the NCBI LinkOut service. At regular intervals (typically every 2-3 months) I upload a dump of the current iPhylo Linkout mapping to the NCBI FTP server and within 48 hours the mapping is incorporated into the NCBI taxonomy pages. These links are labelled "Wikipedia" (see Fig. 4). At the time of writing 53,671 NCBI taxa have a link to a page in Wikipedia.

**Figure:**
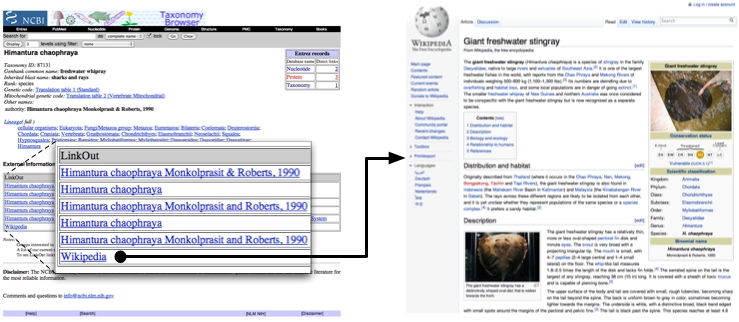
**Fig. 4.** Example of a link between a NCBI taxon page and a page in Wikipedia. In this example, NCBI tax_id 87131  for *Himantura chaophraya* is linked to the page for the Giant freshwater stingray in Wikipedia

Integrating the mapping into Wikipedia has proved less straightforward. Ideally the NCBI tax_id would be stored in the Wikipedia page in a place where it would be easy to add or edit using an automated tool such as a Wikipedia "bot". The Taxobox template would be a good choice as every Wikipedia page for a taxon includes this template. However, a proposal to add NCBI tax_ids to this template generated enough resistance to prevent it being adopted [41]. A possible compromise of adding NCBI tax_ids to the "External links" section of the corresponding Wikipedia pages is being explored.

### Updating the mapping

Because the mapping is implemented as a wiki it is possible to edit an individual NCBI-Wikipedia mapping. Although the user has the option of editing the underlying text of the wiki page, this requires some knowledge of the rather arcane syntax of Mediawiki templates. To make editing easier iPhylo Linkout makes use of the forms feature of Semantic Mediawiki, so that if the user clicks on the "Edit with form" tab they are presented with a simple form to edit (Fig. 5). 

**Figure:**
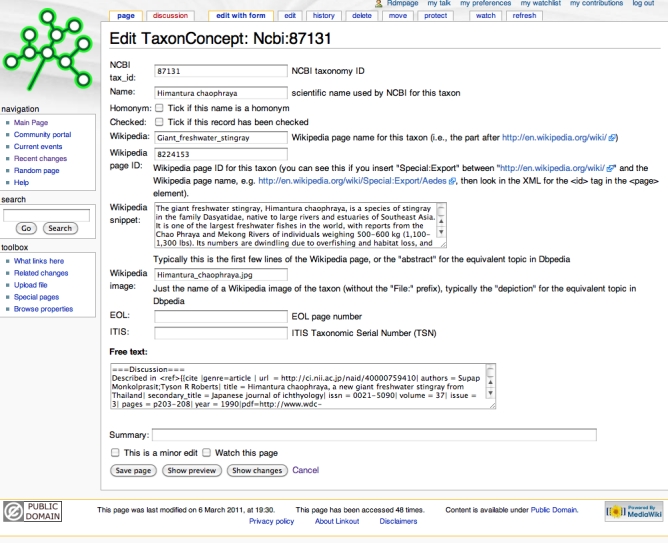
**Fig. 5.** Example of editing a page in iPhylo Linkout using the form interface.

## Future Development

Both NCBI and Wikipedia are continually growing with the sequencing of new taxa and the addition of new articles, respectively. Although the original mapping between NCBI and Wikipedia was based on data from 2009, it has been actively updated manually, or by running local scripts (for example to add large numbers of newly added NCBI tax_ids). An obvious enhancement would be to automate this process. If both the NCBI taxonomy database and Wikipedia both provided services listing recently added or modified taxa, then iPhylo Linkout could consume these and update the mapping automatically. At present neither the NCBI Taxonomy nor Wikipedia explicitly provides such services, although one could be constructed for the NCBI database using their EUtils tools [42]. Wikipedia does provide RSS and IRC feeds of recent changes, but this is for all articles in Wikipedia, rather than the subset of taxon pages. It might be feasible to filter this stream for just those articles that contain a Taxobox.

Although the focus of iPhylo Linkout has been mapping NCBI tax_ids to Wikipedia pages, it could easily be extended to include other identifiers of interest, such as links to the original taxonomic literature. The approach of using a wiki to manage the mapping between identifiers could also be used outside the specific case here of mapping a taxonomic database to Wikipedia pages.

## Availability

The iPhylo Linkout wiki is available at http://iphylo.org/linkout and can be edited by anyone who creates an account. The contents of the mapping are in the public domain.

## Acknowledgements 

Some of the ideas discussed here were developed on my blog [43] and presented at iEvoBio 2010 [44] [45]. I thank Scott Federhen and Kathy Kwan (NCBI) for enabling the upload of NCBI to Wikipedia links to NCBI Linkout, and the numerous participants in the Wikipedia discussion about adding the reverse links to the Taxobox template [41].

## Funding information

This work was funded by the University of Glasgow.

## Competing interests 

The author has declared that no competing interests exist. 
